# Endotoxins are associated with visceral fat mass in type 1 diabetes

**DOI:** 10.1038/srep38887

**Published:** 2016-12-13

**Authors:** Mariann I. Lassenius, Aila J. Ahola, Valma Harjutsalo, Carol Forsblom, Per-Henrik Groop, Markku Lehto

**Affiliations:** 1 Folkhälsan Institute of Genetics, Folkhälsan Research Center, Helsinki, Finland; 2 Abdominal Center Nephrology, University of Helsinki and Helsinki University Central Hospital, Helsinki, Finland; 3 Research Programs Unit, Diabetes and Obesity, University of Helsinki, Helsinki, Finland; 4 National Institute for Health and Welfare, Helsinki, Finland; 5 Baker IDI Heart & Diabetes Institute, Melbourne, Australia

## Abstract

Bacterial lipopolysaccharides (LPS), potent inducers of inflammation, have been associated with chronic metabolic disturbances. Obesity is linked to dyslipidemia, increased body adiposity, and endotoxemia. We investigated the cross-sectional relationships between serum LPS activity and body adiposity as well as inflammation in 242 subjects with type 1 diabetes. Body fat distribution was measured by DXA and serum LPS activity by the limulus amebocyte lysate end-point assay. Since no interaction between visceral fat mass and sex was observed, data were pooled for the subsequent analyses. LPS was independently associated with visceral fat mass, when adjusted for traditional risk factors (age, sex, kidney status, hsCRP, insulin sensitivity). In the multivariate analysis, serum LPS activity and triglyceride concentrations had a joint effect on visceral fat mass, independent of these factors alone. A combination of high LPS and high hsCRP concentrations was also observed in those with the largest visceral fat mass. In conclusion, high serum LPS activity levels were associated with visceral fat mass in subjects with type 1 diabetes strengthening its role in the development of central obesity, inflammation and insulin resistance.

Bacterial endotoxins or lipopolysaccharides (LPS) are potent inducers of systemic inflammation. Endotoxin transport and clearance has been linked to lipoprotein metabolism. Endotoxins are redistributed towards VLDL and LDL particles especially in conditions of lower HDL cholesterol concentrations, like in atherosclerosis and insulin resistance^1^,^2^. Toll-like receptor 4 (TLR4) is a pattern recognition receptor that binds LPS and initiates an inflammatory response. Compared to lean individuals, TLR4 expression is up-regulated in the adipose tissue of obese subjects^3^. A low dose LPS infusion augments adiposity, inflammation, insulin resistance, hyperglycemia and dyslipidemia in mice^4^. In humans, a low dose LPS infusion induces insulin resistance and potentiates the expression of inflammatory markers in adipose tissue^5^.

Studies in non-diabetic children and obese adults, have shown an adverse association between endotoxin levels, central adiposity and insulin resistance^6^,^7^. We have previously shown that a high serum LPS activity is associated with features of the metabolic syndrome and the progression of kidney disease in type 1 diabetes^8^,^9^. Recent studies have further demonstrated a putative link between endotoxins and visceral fat^10^. Excess visceral fat is associated with impaired insulin sensitivity, and the development of vascular complications in diabetes^11^. It has also been suggested that the distribution of body fat could better predict insulin resistance than BMI itself 7,12. Evidently, certain human genetic variants may also have a significant impact on BMI and body adiposity^13^.

Our aim was to investigate the association between serum LPS activity and visceral fat mass, measured by dual-energy x-ray absorptiometry (DXA), in subjects with type 1 diabetes.

## Subjects and Methods

Data from individuals with type 1 diabetes were collected during the Finnish Diabetic Nephropathy (FinnDiane) Study visits taking place in Helsinki between years 2011 and 2016. In the current analyses, we included data from all individuals with completed LPS and body composition measurements, and either normal urinary albumin excretion rate (T1D-normo, n = 156), microalbuminuria (T1D-micro, n = 42) or macroalbuminuria (T1D-macro, n = 46). Renal status was assessed by the albumin excretion rate (AER) in two out of three consecutive timed urine collections using the following criteria: normal AER <20 μg/min or <30 mg/24 h, and macroalbuminuria ≥200 μg/min or ≥300 mg/24 h. Kidney transplant recipients and those undergoing dialysis were excluded from the analysis. Type 1 diabetes was defined as disease onset before the age of 40 and the initiation of insulin injections within one year of diagnosis.

Body composition was measured by DXA (Lunar iDXA by GE Healthcare, Scanex Medical Systems, Finland). The iDXA software calculates the body fat distribution including visceral-, android-, and gynoid fat mass. BMI was calculated (weight/height^2^). Insulin sensitivity was assessed calculating the estimated glucose disposal rate (eGDR): eGDR = 24.4 − 12.97 × WHR − 3.39 × AHT − 0.60 × A1C, where WHR stands for waist-to-hip ratio and AHT for antihypertensive treatment and/or blood pressure ≥140/90 mmHg (yes = 1, no = 0). Daily insulin dose was self-reported. Serum lipid concentrations were analyzed centrally by automated enzymatic methods (Hoffmann-La Roche, Basel, Switzerland). Serum high-sensitive C-reactive protein (hsCRP) concentration was measured by immunoassay (Modular analyzer, Roche). LPS activity was measured by the Limulus amebocyte lysate (LAL) assay from 1:5 diluted fasting serum samples (Hycult Biotechnology, Uden, the Netherlands). For the LAL assay, inter- and intra-assay coefficients of variation were 16.1% and 4.5%, respectively. The sensitivity limit for the assay was 0.02 EU/ml.

### Statistical analyses

Normality of variable distribution was assessed with the Kolmogorov-Smirnov test. Normally distributed variables are reported as mean ± standard deviation, non-normally distributed variables as median [25^th^–75^th^ quartile]. Correlation coefficients were calculated by Spearman or Pearson’s correlation test as appropriate. Differences in frequencies were assessed by Pearson’s Chi squared analysis. Differences in variation between groups were calculated using Mann-Whitney U-test, Kruskal-Wallis test and ANOVA, as appropriate. The population was divided into tertiles based on serum LPS activity, triglyceride-, or hsCRP concentrations to further explore the role of endotoxins, dyslipidemia, and inflammation on visceral fat mass.

The interaction between LPS and gender on visceral fat mass was assessed using a generalized linear model. Since no interaction was evident, all data were analyzed together. The interaction between LPS and triglyceride concentration on visceral fat mass was analyzed similarly. As LPS and triglyceride concentration showed significant interaction, the interaction term was included in the multivariable model. Because LPS and triglycerides are highly correlated, we furthermore visualized their relative relationship using the generalized additive modeling (GAM) without *a priori* assumptions of the shape of the relation. The GAM modelling allows the inclusion of non-parametric smoothing functions to identify a potential non-linearity in the relationship between the independent and the dependent variables^14^,^15^. The generalized cross-validation function (GCV) was used as a criterion for selection of the smoothing parameters to determine an appropriate level of smoothing. Analyses were carried out using IBM SPSS Statistics for Windows, Version 22.0 (IBM Corp, Armonk, NY, USA), and R open source software (http://www.r-project.org). GAM models were fitted using the mgcv library in R^16^.

### Ethics

The study protocol was approved by the Ethics Committee of the Hospital District of Helsinki and Uusimaa. The study was carried out in accordance with the approved guidelines. Participants gave their written informed consent prior to participating in the study.

## Results

Patient characteristics divided by renal status are shown in Table 1. The proportion of men was highest among T1D-macro. The diabetes duration and visceral fat mass increased with worsening renal status. Insulin sensitivity (eGDR) was highest in T1D-normo. No differences were observed in HbA_1c_, HDL cholesterol concentration, LPS activity, insulin dose, use of insulin pump, BMI or hsCRP concentration amongst the three groups.

**Figure 1:**
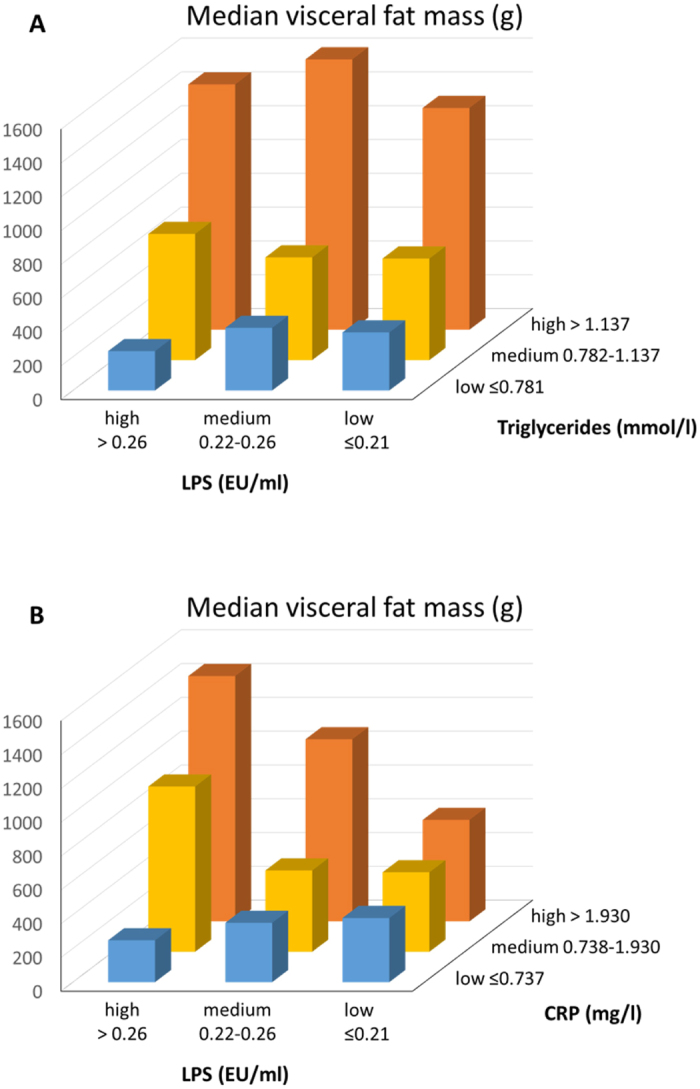
(**A**) Visceral fat mass (g) according to LPS and triglyceride tertiles (low/medium/high) in subjects with type 1 diabetes. Higher visceral fat mass was observed in those with a combination of high LPS activity/high triglyceride concentration (N = 50) compared to those with low LPS activity/low triglyceride concentration (N = 43, p < 0.001). **(B)** Visceral fat mass (g) according to LPS and hsCRP tertiles (low/medium/high) in subjects with type 1 diabetes. Higher visceral fat mass was observed in those with a combination of high LPS activity/high hsCRP concentration (N = 32) compared to low LPS activity/low hsCRP concentration (N = 38, p < 0.001).

Serum LPS activity correlated positively with BMI (r = 0.274, p < 0.001), visceral fat mass (r = 0.248, p < 0.001), android to gynoid fat ratio (r = 0.231, p < 0.001), serum triglyceride concentration (r = 0.477, p < 0.001), and hsCRP concentration (r = 0.276, p < 0.001). Negative correlation was observed between insulin dose and eGDR (r = −0.205, p = 0.002). Supplementary Figure S1a and S1b show that triglyceride concentration seems to overrun the effect of LPS on the visceral fat mass. However, the relationship between LPS and triglycerides is complicated and interaction between LPS and triglyceride concentration was found, indicating that LPS has an effect on certain levels of triglyceride concentration. Therefore, the effect of serum LPS activity and triglyceride concentration on visceral fat mass were further explored in LPS and triglyceride tertiles (low/medium/high). Subjects in the high triglyceride tertile presented the highest visceral fat mass. Notably, within each LPS tertile, the visceral fat mass was observed to increase towards the higher TG tertiles (p < 0.001, all) (Fig. 1A). Moreover, the visceral fat mass among those with high LPS activity and high triglyceride level was higher compared to the group with both low LPS activity and low triglyceride level (high/high vs. low/low, median [IQR]: 1452 [767–2001] vs. 343 [144–515] g, p < 0.001). The Supplementary Figure S2 provides a deeper view on the interaction effect of LPS and triglyceride levels on the visceral fat mass focusing on the 75% of the population in the area with the densest accumulation of observations. Similarly, subjects in the high-LPS/high-hsCRP tertile presented greater visceral fat mass compared to those with low-LPS/low-hsCRP concentrations (median [IQR]: 1452 [813–2014] vs. 379 [156–818] g, p < 0.001) (Fig. 1B).

The independent association between LPS activity and visceral fat mass was analyzed in a number of multivariable models (Table 2). With Model 1, we aimed at looking at this association while taking inflammation into account. In this model, LPS activity was positively associated with the visceral fat mass (B = 1253.2, 95% Wald Confidence Interval 386.4–2120.0, Wald Chi-Squared 8.029, p = 0.005). Further adjusting for insulin sensitivity (eGDR), LPS remained a significant predictor of visceral fat mass (Model 2: B = 848.6, 95% Wald CI 48.4–1648.8, Wald Chi Squared 4.320, p = 0.038). However, after further adjusting for triglyceride concentration (Model 3), LPS no longer predicted visceral fat mass. To the final model (Model 4) we incorporated a LPS*triglyceride interaction term, which was significantly associated with the visceral fat mass (B = −634.2, 95% Wald CI −1092.6–−175.7, Wald Chi-Squared 7.350, p = 0.007), independently of the two factors entered in the model separately.

## Discussion

Inflammation and insulin resistance are intertwined processes that are strongly linked to overweight and intra-abdominal obesity. We observed that serum LPS activity is associated with visceral fat mass in type 1 diabetes, independent of the traditional risk factors such as age, insulin sensitivity, and inflammation.

High serum LPS activity has been associated with central obesity and insulin resistance, and it has also been shown to predict the development of type 2 diabetes^6^,^17^. Intestinal and oral microbial communities are the most likely sources of bacterial endotoxins in humans. Frequent use of antibiotics may have adverse effects on gut homeostasis accompanied by dysbiosis and increased intestinal permeability of bacterial compounds. Childhood hospitalizations related to infections have been associated with an increased risk of obesity and metabolic syndrome in adulthood^18^. In conditions of intestinal inflammation, such as Crohn’s disease, visceral fat accumulation is evident^19^.

Dietary fat intake may increase systemic LPS activity, driving adipose inflammation^20^. In a recent study, circulating LPS activity levels correlated with intra-abdominal fat volumes, but only modestly with the subcutaneous fat volumes in patients undergoing bariatric surgery. Notably, a significant decrease in serum endotoxin levels was still evident one year after the operation^21^. Higher CRP levels may arise through a local inflammation of the adipose tissue associated with an increased bacterial burden^19^.

Serum triglycerides and LPS are strongly correlated^9^. Indeed, we observed an interaction between LPS and triglycerides on visceral fat mass. Importantly, the interaction term was independent of the individual factors alone, indicating that the combination of high serum LPS and triglycerides have additive effects on visceral fat mass. This observation is in concordance with previous rat studies, showing that a low dose intraperitoneal LPS injection increases liver triglyceride production, while high doses decrease triglyceride-rich lipoprotein clearance^22^. Moreover, in healthy volunteers, a small intravenous dose of LPS acutely increased serum triglyceride levels^23^, while in mice a subcutaneous infusion of LPS for four weeks significantly increased visceral fat mass^4^. Cross-sectional nature and small study population are potential limitations of the current study. Moreover, due to the exclusion of individuals with end-stage renal disease, the results may not be generalizable to these individuals. Prospective studies are needed to establish the causalities related to the observations.

In conclusion, independent of traditional risk factors, serum LPS activity is associated with visceral fat mass in type 1 diabetes. The LPS-association with visceral fat is pronounced at high triglyceride concentrations. This strengthens the hypothesis that both bacterial endotoxins and dyslipidemia contribute to the development of central obesity, inflammation and insulin resistance and thus may subsequently be associated with adverse cardio-metabolic outcomes.

## Additional Information

**How to cite this article:** Lassenius, M. I. *et al*. Endotoxins are associated with visceral fat mass in type 1 diabetes. *Sci. Rep.* **6**, 38887; doi: 10.1038/srep38887 (2016).

**Publisher's note:** Springer Nature remains neutral with regard to jurisdictional claims in published maps and institutional affiliations.

## Supplementary Material

Supplementary Information

## Figures and Tables

**Table 1:** Clinical characteristics of study participants divided by renal status.

	Normal AER n = 154	Microalbuminuria n = 42	Macroalbuminuria n = 46	p
Men, n (%)	66 (43)	19 (45)	31 (67)	0.013
Age (years)	48.3 ± 12.7	49.0 ± 11.4	52.7 ± 8.2	0.085
Diabetes duration (years)	31.1 ± 12.7	35.9 ± 9.6	38.4 ± 9.7	<0.001
HbA_1c_, mmol/mol (%)	65 ± 12 (8.1 ± 1.1)	68 ± 13 (8.4 ± 1.1)	66 ± 13 (8.2 ± 1.3)	0.241
Cholesterol (mmol/l)	4.5 (3.9–5.0)	4.7 (4.2–5.2)	4.2 (3.7–4.8)	0.046
HDL cholesterol (mmol/l)	1.58 (1.36–1.94)	1.54 (1.20–2.01)	1.41 (1.12–1.85)	0.157
Triglycerides (mmol/l)	0.88 (0.67–1.16)	1.05 (0.70–1.76)	1.06 (0.84–1.77)	<0.001
LPS (EU/ml)	0.24 (0.20–0.28)	0.24 (0.20–0.31)	0.24 (0.20–0.31)	0.942
Insulin dose (IU/kg)	0.54 (0.43–0.71)	0.65 (0.49–0.76)	0.54 (0.47–0.74)	0.193
Insulin pump, n (%)	32 (21)	10 (24)	15 (33)	0.252
BMI (kg/m^2^)	24.9 (22.8–27.6)	26.5 (23.0–30.3)	25.5 (23.1–28.7)	0.087
eGDR (mg/kg/min)	5.4 (4.0–8.3)	3.8 (3.0–5.1)	3.9 (2.8–4.7)	<0.001
hsCRP (mg/l)	1.11 (0.51–2.47)	1.63 (0.79–3.06)	1.01 (0.49–3.52)	0.391
Visceral fat mass all (g)	452 (194–1022)	737 (381–2020)	1096 (471–1881)	<0.001
Visceral fat mass, men (g)	724 (372–1511)	1298 (532–3039)	1100 (728–1932)	0.044
Visceral fat mass, women (g)	294 (140–737)	523 (300–1816)	644 (236–1556)	0.017
Android fat mass, all (g)	1686 (1131–2924)	2292 (1456–4018)	2243 (1394–3321)	0.012
Android fat mass, men (g)	1913 (1189–2967)	3047 (1470–4264)	2302 (1499–3028)	0.129
Android fat mass, women (g)	1569 (1096–2412)	1958 (1417–3809)	1960 (759–3838)	0.149
Gynoid fat mass, all (g)	3642 (2945–4735)	4089 (3333–5303)	3689 (2722–4590)	0.267
Gynoid fat mass, men (g)	3343 (2573–3907)	3715 (2466–4889)	3557 (2735–4170)	0.407
Gynoid fat mass, women (g)	4300 (3210–5150)	4360 (3517–5857)	4368 (2222–5496)	0.556
Android/Gynoid ratio, all (g)	0.45 (0.33–0.61)	0.58 (0.40–0.75)	0.63 (0.49–0.78)	<0.001
Android/Gynoid ratio, men (g)	0.57 (0.44–0.80)	0.75 (0.50–0.95)	0.69 (0.53–0.84)	0.077
Android/Gynoid ratio, women (g)	0.38 (0.30–0.51)	0.50 (0.35–0.66)	0.49 (0.33–0.71)	0.028

Data are presented as frequency (percentage) for categorical variables, mean ± SD for normally distributed continuous variables, and median (25^th^–75^th^ quartile) for non-normally distributed continuous variables. Significance across the three groups has been studied with Chi squared, ANOVA and Kruskal-Wallis test, respectively. AER, albumin excretion rate; LPS, lipopolysaccharides; eGDR, estimated glucose disposal rate; hsCRP, high-sensitive C-reactive protein.

**Table 2:** Factors associated with visceral fat mass.

		B	95% Wald Confidence Interval	Wald Chi-squared	*p*
Model 1	LPS activity	1253.2	386.4–2120.0	8.029	0.005
	Women	−595.1	−810.5–−379.6	29.303	<0.001
	Age	12.0	3.1–20.9	7.038	0.008
	Nephropathy	211.4	−52.6–475.4	2.463	0.117
	hsCRP	64.7	38.4–91.0	23.299	<0.001
Model 2	LPS activity	848.6	48.4–1648.8	4.320	0.038
	Women	−309.8	−524.6–−95.1	7.995	0.005
	Age	4.9	−3.4–13.3	1.344	0.246
	Nephropathy	−19.0	−269.8–231.8	0.022	0.882
	hsCRP	32.8	6.9–58.6	6.174	0.013
	eGDR	−158.0	−205.9–−110.1	41.763	<0.001
Model 3	LPS activity	−678.9	−1558.8–201.1	2.286	0.131
	Women	−171.5	−373.5–30.6	2.766	0.096
	Age	6.1	−1.6–13.8	2.392	0.122
	Nephropathy	0.1	−230.3–230.6	0.000	0.999
	hsCRP	10.1	−14.7–34.9	0.635	0.426
	eGDR	−126.7	−171.8–−81.6	30.292	<0.001
	Triglycerides	452.6	309.3–596.0	38.295	<0.001
Model 4	LPS activity	453.8	−737.1–1644.8	0.558	0.455
	Women	−156.4	−355.3–42.5	2.376	0.123
	Age	5.4	−2.2–13.0	1.959	0.162
	Nephropathy	1.3	−225.2–227.7	0.000	0.991
	hsCRP	5.0	−19.7–29.7	0.158	0.691
	eGDR	−125.0	−169.3–−80.6	30.519	<0.001
	Triglycerides	732.7	486.0–979.4	33.892	<0.001
	LPS*TG interaction	−634.2	−1092.6–−175.7	7.350	0.007

Generalized linear model. LPS, lipopolysaccharides; hsCRP, high-sensitivity C-reactive protein; eGDR, estimated glucose disposal rate; TG, triglycerides.
