# Supplementary material for: Endotoxins are associated with visceral fat mass in type 1 diabetes
Source: Sci Rep. 2016 Dec 13;6:38887. doi: 10.1038/srep38887 (PMC5153626; doi:10.1038/srep38887)
Supplement: Supplementary Information [file srep38887-s1.pdf]

Endotoxins are associated with visceral fat mass in type 1 diabetes

Mariann I Lassenius†[1,2,3], Aila J Ahola†[1,2,3], Valma Harjutsalo[1,2,3,4], Carol Forsblom[1,2,3], Per-Henrik Groop[1,2,3,5], Markku Lehto*[1,2,3]

Figure S1. Shape of association drawn from the GAM analyses between A) serum LPS activity and visceral fat mass and B) triglyceride levels and visceral fat mass. Grey areas show 95% confidence intervals.

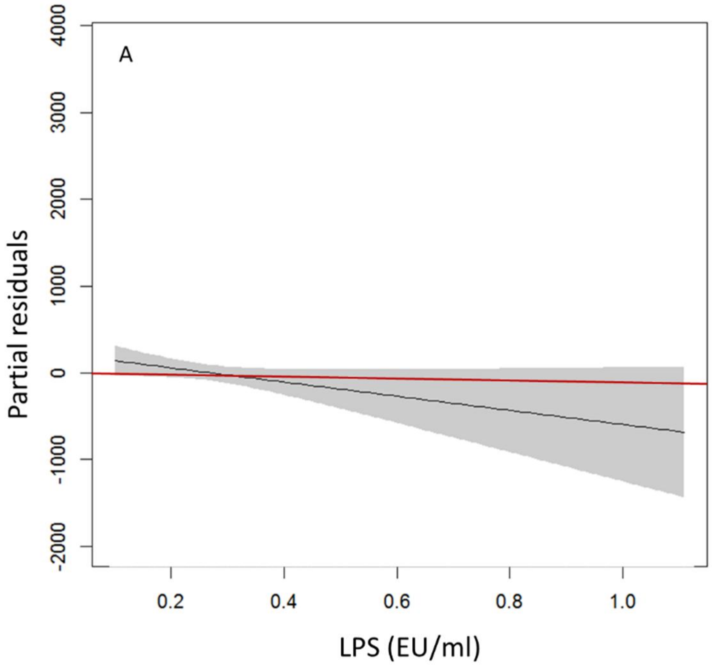

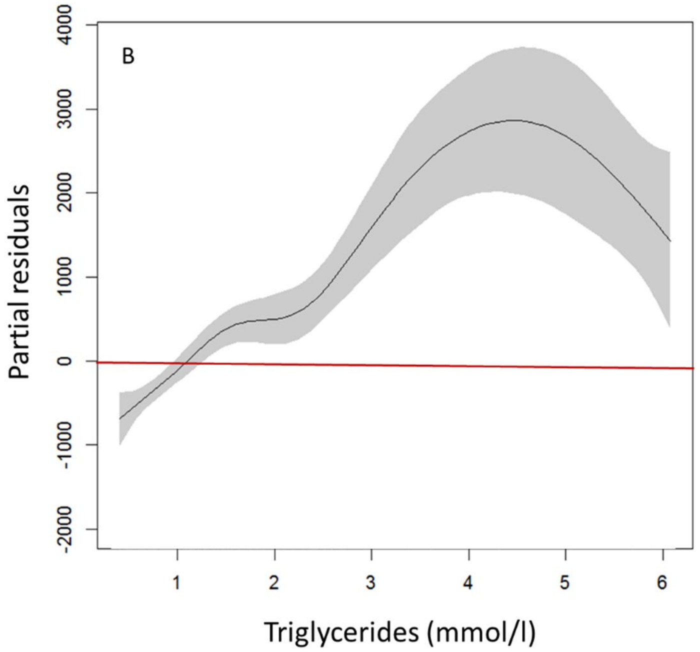

Figure S2. Visceral fat mass (g) according to serum LPS activity and triglyceride levels in 75% of study population.

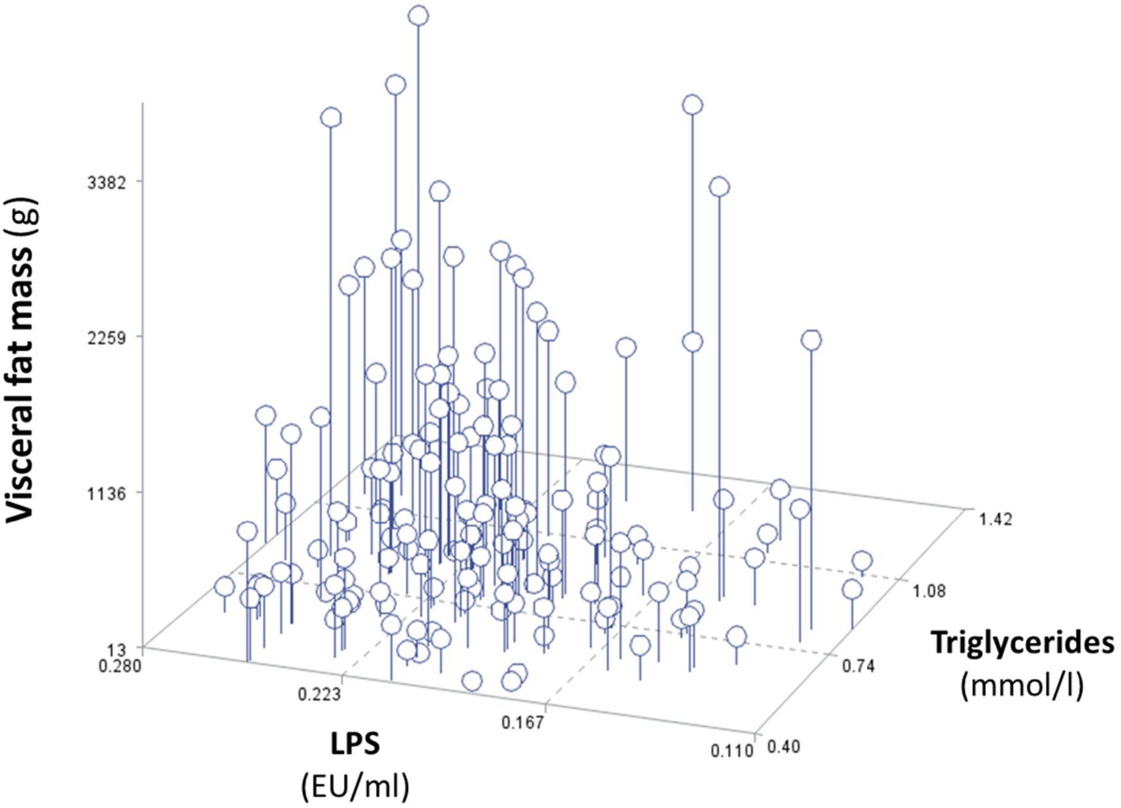

The physicians and nurses at each centre participating in the collection of patients:

Anjalankoski Health Centre: S. Koivula and T. Uggeldahl; Central Finland Central Hospital: T. Forslund, A. Halonen, A. Koistinen, P. Koskiaho, M. Laukkanen, J. Saltevo and M. Tiihonen; Central Hospital of Åland Islands: M. Forsen, H. Granlund, A.-C. Jonsson and B. Nyroos; Central Hospital of Kanta-Häme: P. Kinnunen, A. Orvola, T. Salonen and A. Vähänen; Central Hospital of Kymenlaakso: R. Paldanius, M. Riihelä and L. Ryysy; Central Hospital of Länsi-Pohja: H. Laukkanen, P. Nyländen and A. Sademies; Central Ostrobothnian Hospital District: S. Anderson, B. Asplund, U. Byskata, P. Liedes, M. Kuusela and T. Virkkala; City of Espoo Health Centre (Espoonlahti): A. Nikkola and E. Ritola; (Tapiola): M. Niska and H. Saarinen; (Viherlaakso): A. Lyytinen; City of Helsinki Health Centre (Puistola): H. Kari and T. Simonen; (Suutarila): A. Kaprio, J. Kärkkäinen and B. Rantaeskola; (Töölö): P. Kääriäinen, J. Haaga and A-L. Pietiläinen; City of Hyvinkää Health Centre: S. Klemetti, T. Nyandoto, E. Rontu and S. Satuli-Autere; City of Vantaa Health Centre (Korso): R. Toivonen and H. Virtanen; (Länsimäki): R. Ahonen, M. Ivaska-Suomela and A. Jauhiainen; (Martinlaakso): M. Laine, T. Pellonpää and R. Puranen; (Myyrmäki): A. Airas, J. Laakso and K. Rautavaara; (Rekola): M. Erola and E. Jatkola; (Tikkurila): R. Lönnblad, A. Malm, J. Mäkelä and E. Rautamo; Heinola Health Centre: P. Hentunen and J. Lagerstam; Helsinki University Central Hospital (Department of Medicine, Division of Nephrology): D. Cordin, J. Fagerudd, M. Feodoroff, O. Heikkilä, L. Kyllönen, J. Kytö, K. Pettersson-Fernholm, M. Rosengård-Bärlund, M. Rönnback, L. Thorn and J. Wadén; Herttoniemi Hospital: V. Sipilä; Hospital of Lounais-Häme: T. Kalliomäki, J. Koskelainen, R. Nikkanen, N. Savolainen, H. Sulonen and E. Valtonen; Iisalmi Hospital: E. Toivanen; Jokilaakso Hospital: A. Parta and I. Pirttiniemi; Jorvi Hospital: S. Aranko, S. Ervasti, R. Kauppinen-Mäkelin, A. Kuusisto, T. Leppälä, K. Nikkilä and L. Pekkonen; Jyväskylä Health Centre: K. Nuorva and M. Tiihonen; Kainuu Central Hospital: S. Jokelainen, P. Kemppainen, A-M. Mankinen and M. Sankari; Kerava Health Centre: H. Stuckey and P. Suominen; Kirkkonummi Health Centre: A. Lappalainen, M. Liimatainen and J. Santaholma; Kivelä Hospital: A. Aimolahti and E. Huovinen; Koskela Hospital: V. Ilkka and M. Lehtimäki; Kotka Health Centre: E. Pälikkö-Kontinen and A. Vanhanen; Kouvola Health Centre: E. Koskinen and T. Siitonen; Kuopio University Hospital: E. Huttunen, R. Ikäheimo, P. Karhapää, P. Kekäläinen, M. Laakso, T. Lakka, E. Lampainen, L. Moilanen, L. Niskanen, U. Tuovinen, I. Vauhkonen and E. Voutilainen; Kuusamo Health Centre: T. Kääriäinen and E. Isopoussu; Kuusankoski Hospital: E. Kilkki, I. Koskinen and L. Riihelä; Laakso Hospital, Helsinki: T. Meriläinen, P. Poukka, R. Savolainen and N. Uhlenius; Lahti City Hospital: A. Mäkelä and M. Tanner; Lapland Central Hospital: L. Hyvärinen, S. Severinkangas and T. Tulokas; Lappeenranta Health Centre: P. Linkola and I. Pulli; Lohja Hospital: T. Granlund, M. Saari and T. Salonen; Länsi-Uusimaa Hospital: I.-M. Jousmaa and J. Rinne; Loimaa Health Centre: A. Mäkelä

and P. Eloranta; Malmi Hospital: H. Lanki, S. Moilanen and M. Tilly-Kiesi; Mikkeli Central Hospital: A. Gynther, R. Manninen, P. Nironen, M. Salminen and T. Vänttinen; Mänttä Regional Hospital: I. Pirttiniemi and A-M. Hänninen; North Karelian Hospital: U-M. Henttula, P. Kekäläinen, M. Pietarinen, A. Rissanen and M. Voutilainen; Nurmijärvi Health Centre: A. Burgos and K. Urtamo; Oulaskangas Hospital: E. Jokelainen, P.-L. Jylkkä, E. Kaarlela and J. Vuolaspuro; Oulu Health Centre: L. Hiltunen, R. Häkkinen and S. Keinänen-Kiukaanniemi; Oulu University Hospital: R. Ikäheimo; Päijät-Häme Central Hospital: H. Haapamäki, A. Helanterä, S. Hämäläinen, V. Ilvesmäki and H. Miettinen; Palokka Health Centre: P. Sopanen and L. Welling; Pieksämäki Hospital: V. Javtsenko and M. Tamminen; Pietarsaari Hospital: M-L. Holmbäck, B. Isomaa and L. Sarelin; Pori City Hospital: P. Ahonen, P. Merensalo and K. Sävelä; Porvoo Hospital: M. Kallio, B. Rask and S. Rämö; Raahe Hospital: A. Holma, M. Honkala, A. Tuomivaara and R. Vainionpää; Rauma Hospital: K. Laine, K. Saarinen and T. Salminen; Riihimäki Hospital: P. Aalto, E. Immonen and L. Juurinen; Salo Hospital: A. Alanko, J. Lapinleimu, P. Rautio and M. Virtanen; Satakunta Central Hospital: M. Asola, M. Juhola, P. Kunelius, M.-L. Lahdenmäki, P. Pääkkönen and M. Rautavirta; Savonlinna Central Hospital: T. Pulli, P. Sallinen, M. Taskinen, E. Tolvanen, H. Valtonen and A. Vartia; Seinäjoki Central Hospital: E. Korpi-Hyövälti, T. Latvala and E. Leijala; South Karelia Central Hospital: T. Ensala, E. Hussi, R. Härkönen, U. Nyholm and J. Toivanen; Tampere Health Centre: A. Vaden, P. Alarotu, E. Kujansuu, H. Kirkkopelto-Jokinen, M. Helin, S. Gummerus, L. Calonius, T. Niskanen, T. Kaitala and T. Vatanen; Tampere University Hospital: I. Ala-Houhala, T. Kuningas, P. Lampinen, M. Määttä, H. Oksala, T. Oksanen, K. Salonen, H. Tauriainen and S. Tulokas; Tiirismaa Health Centre: T. Kivelä, L. Petlin and L. Savolainen; Turku Health Centre: I. Hämäläinen, H. Virtamo and M. Vähätalo; Turku University Central Hospital: K. Breitholz, R. Eskola, K. Metsärinne, U. Pietilä, P. Saarinen, R. Tuominen and S. Äyräpää; Vaajakoski Health Centre: K. Mäkinen and P. Sopanen; Valkeakoski Regional Hospital: S. Ojanen, E. Valtonen, H. Ylönen, M. Rautiainen and T. Immonen; Vammala Regional Hospital: I. Isomäki, R. Kroneld and M. Tapiolinna-Mäkelä; Vaasa Central Hospital: S. Bergkulla, U. Hautamäki, V.-A. Myllyniemi and I. Rusk.
